# Molecular PET Imaging of Cyclophosphamide Induced Apoptosis with ^18^F-ML-8

**DOI:** 10.1155/2015/317403

**Published:** 2015-04-09

**Authors:** Shaobo Yao, Kongzhen Hu, Ganghua Tang, Siyuan Gao, Caihua Tang, Baoguo Yao, Dahong Nie, Ting Sun, Shende Jiang

**Affiliations:** ^1^Department of Nuclear Medicine, The First Affiliated Hospital, Sun Yat-sen University, Guangzhou 510080, China; ^2^School of Pharmaceutical Science and Technology, Tianjin University, Tianjin 300072, China; ^3^Department of Cardiology, Shanghai Ninth People's Hospital, Shanghai Jiao Tong University School of Medicine, Shanghai 200011, China

## Abstract

In this paper, a novel small-molecular apoptotic PET imaging probe, ^18^F-ML-8 with a malonate motif structure, is presented and discussed. After study, the small tracer that belongs to a member of ApoSense family is proved to be capable of imaging merely apoptotic regions in the CTX treated tumor-bearing mice. The experimental result is further confirmed by in vitro cell binding assays and TUNEL staining assay. As a result, ^18^F-ML-8 could be used for noninvasive visualization of apoptosis induced by antitumor chemotherapy.

## 1. Introduction

Apoptosis is a fundamental biologic process that could be described as controlled demolition of cell and is inherent in every cells and plays vital roles in numerous medical disorders [1, 2]. Great majority of common chemotherapy agents trigger tumor cell death by induction of apoptosis [3]. Apoptosis could result in pathologic alteration (such as neurodegenerative diseases) and could be triggered by therapeutic treatment such as irradiation or chemotherapy [4, 5]. In vivo imaging of this process is focusing the attention for many years, especially the assessment of therapeutic response in oncology [2, 6–10]. Consequently, noninvasive, functional, and molecular imaging of apoptosis may be of great value in the future clinical practice for the diagnosis of disease or evaluation of treatment efficacy [2, 11, 12].

**Figure 1:**
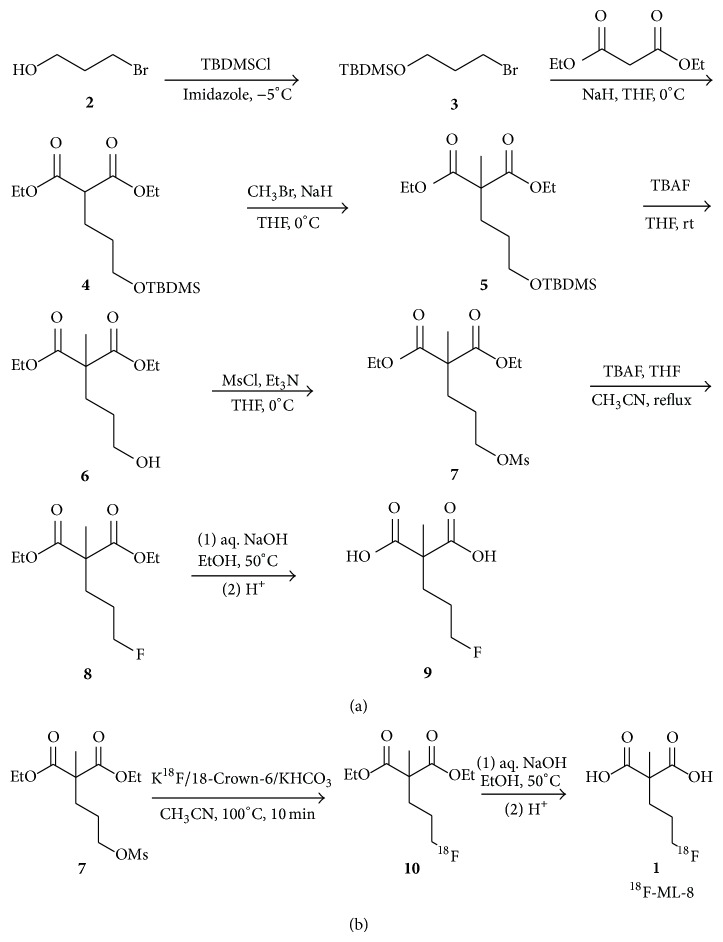
(a) Chemical synthesis of ML-8 (**9**) and its precursor (**7**) starting from 3-bromo-propanol and diethyl malonate. (b) Radiosynthesis of ^18^F-ML-8 from the precursor (**7**). O-TBDMS = O-*tert* butyl dimethyl silyl; O-Et = O-ethyl; O-Ms = O-mesyl.

Approaches for imaging apoptosis have been studied with radiolabeled Annexin V, synaptotagmin, and caspase substrates with large and small molecules; however, each has limitations or drawbacks. For example, the labeled Annexin V has unfavourable pharmacokinetics and immunogenicity in vivo, which hinders its further clinical trial. The radiolabelled caspase substrates show unfavourable pharmacokinetics and cannot differentiate apoptotic cells from necrotic cells [13–15]. In order to overcome the limitations of large molecules our research group has developed a low molecular weight ^18^F-labeled polypeptide tracer ^18^F-FPDuramycn, which is proven to be a potential PET agent for imaging of cell death [16]. However, limitations and drawbacks such as suboptimal biodistribution or pharmacokinetics, relatively low binding ability, and long radiosynthesis time badly blocked the clinical application of ^18^F-FPDuramycn. In recent years, a novel series of low molecular weight compounds were named ApoSense family and had been widely studied for in vivo detection and imaging of apoptosis or cell death [17]. These categories of small molecules were sensitive to the alterations in plasma membrane and phospholipid scrambling. The scrambling process was the hallmark of early apoptotic process in mammalian cellular membranes and could be mediated, at least in part, by the scramblase proteins [17, 18]. Among them, ^18^F-ML-10, with a malonate motif structure, was the most representative and prospective radiopharmaceuticals that could distinguish apoptotic cells from necrotic cells [19]. However, ^18^F-ML-10 also exhibited some limitations, such as the relatively low accumulation in anticancer chemotherapeutic drug treated tumor cells. Therefore, we designed and synthesized ^18^F-labeled-3-fluoropentyl-2-methyl-malonic acid (^18^F-ML-8, Figure 1), an analogue to ^18^F-ML-10 with lower molecular weight (MW 178) than ^18^F-ML-10.

In this paper, we present the chemical synthesis of the standard ML-8 and its precursor, ^18^F-radiosynthesis of ^18^F-ML-8, and metabolism and biodistribution studies in normal mice. In vivo small-animal PET imaging and assessment of apoptosis due to antitumor chemotherapy are also discussed in detail.

## 2. Materials and Methods

### 2.1. General

All chemicals obtained commercially were of analytical grade (Sigma-Aldrich, Milwaukee, WI, USA) and used without further purification. Sep-Pak light QMA and Sep-Pak plus C18 cartridges were obtained from Waters Corporation (Milford, MA, USA). SEP-PAK light QMA cartridges were preconditioned with NaHCO_3_ aqueous (8.4%) and water before use. Sep-Pak plus C18 cartridges were preconditioned with ethanol and water in advance.

^1^H nuclear magnetic resonance (^1^H NMR) spectra were obtained with a Viarian INOVA (400 MHz or 600 MHz) spectrometer (Bruker, Germany). Chemical shifts are reported as per million (*δ* ppm) using tetramethylsilane (TMS) as internal standard or by reference to proton resonances resulting from incomplete deuteration of the NMR solvent. Coupling constants are reported in hertz (Hz) and multiplicity was abbreviated as s (singlet), d (doublet), t (triplet), br (broad), m (multiplet), and dt (double of triplet). Flash chromatography was performed on silica gel (300–400 mesh). Thin layer chromatographies (TLC) were run on precoated aluminium plates (Kieselgel 60 F254, Merk, Germany) and visualized with UV light or basic aqueous potassium permanganate color developing agent.

The PET-MF-2V-IT-I synthesis module was purchased from Beijing PET Company (Beijing, China). Analytic HPLC was performed using a Agilent 1200 Series analytical HPLC system (Agilent Technologies, Palo Alto, CA, USA) equipped with a Agilent Series HPLC pump (with the flow rate of 1 mL/min), UV detector (Agilent interface 35900E, Agilent Technologies, USA) at 211 nm, and a B-FC-3200 high energy PMT Detector (Bioscan Inc., Washington, DC, USA). Radioactivity was measured by a CRC-25PET Radioisotope Dose Calibrator (Capintec, Inc., Ramsey, NJ, USA).

### 2.2. Chemical Synthesis

The synthesis of 2-(3-fluoropropyl)-2-methyl-malonic acid (ML-8) was performed by a similar method as previously described [19]. Detailed procedure was included in the Supplementary Materials (see Supplementary Materials available online at http://dx.doi.org/10.1155/2015/317403).

### 2.3. Radiosynthesis Assays

The overall yield of the chemical synthesis of the precursor, ML-8-dimesylate, was about 72%, a high yield over five steps. ^18^F-ML-8 was synthesized from its respective precursor by a nucleophilic substitution reaction (Figure 1(b)). ^18^F-fluoride delivered from a cyclotron was trapped on an ion exchange resin (Waters QMA light, ABX) and excess ^18^H_2_O was removed and eluted with 18-Crown-6 (14 mg, 53 *μ*mol) (Sigma-Aldrich) and potassium bicarbonate (KHCO_3_) (3 mg, 30 *μ*mol) complex. The complex was azeotropic drying with CH_3_CN at 100°C under a nitrogen stream. Then a solution of ML-8 precursor** 7** (5 mg, 28 *μ*mol) in anhydrous CH_3_CN (1 mL) was added and the fluorination was carried out at 100°C for 12 min. After the fluorination, aliquots (5 *μ*L) were taken and subjected to radio-HPLC analysis (CH_3_CN/H_2_O/TFA, 50/50/0.1 v/v/v). After quenched and diluted with water (0.1% TFA, 10 mL), Sep-Pak C18 cartridge (Waters) was employed to purify ethyl-protected ^18^F-ML-8. After elution with ethanol (3 mL), the solution was evaporated to dryness under a stream of nitrogen at 80°C. The hydrolysis of ester was accomplished by addition of NaOH (1 N, 0.1 mL) and carried out at 50°C. Aliquots (2 *μ*L) were taken at 5, 10, and 15 min, diluted with CH_3_CN, and subjected to radio-HPLC analysis (CH_3_CN/H_2_O/TFA, 30/70/0.1 v/v/v). The identity of compound** 1** was confirmed by HPLC coinjection with the standard. HCl (aq. 1 N) was added to the vessel to adjust pH 5-6. Sterile water was added and the formulated mixture was filtered through a sterile Millipore filter (0.22 *μ*m, 4 mm) directly into a sterile product vial (10 mL size).

### 2.4. Animal Models and Treatment Plan

All animals were conducted in accordance with legal and ethical guidelines and recommendations of the Committee on Animal and Human Research at the First Affiliated Hospital, Sun Yat-sen University. The protocol was fully approved by the local institutional review committee on animal care. The SPCA-1 tumor models were generated by subcutaneous injection of 5 × 10^6^ human lung adenocarcinoma cells into the right shoulder of female athymic nude mice (Laboratory Animal Center of Sun Yat-sen University). Tumor sizes were monitored with a vernier caliper. When the tumor was about 0.5–1.0 cm in diameter, ten allografted mice were randomized into two groups. One group (*n* = 5) were treated with the intraperitoneal injection of cyclophosphamide (CTX, 100 mg/kg) to perform the therapeutic induced cell death within an interval 1 day. The other group (*n* = 5) received saline only. The two groups of nude mice were performed by small-animal PET imaging separately with ^18^F-ML-8.

### 2.5. In Vitro and In Vivo Stability

For in vitro assays, samples of ^18^F-ML-8 0.1 mL (1.85 MBq, 50 *μ*Ci) dissolved in sterile saline were incubated with 0.2 mL of mouse serum at 37°C with gentle shaking. An aliquot of the serum sample was analyzed using the above analytical HPLC system to determine the percentage of intact ^18^F-ML-8 in 30, 60, 90, and 120 min.

For in vivo stability tests, mice were injected intravenously with a dosage of 11.1 MBq (300 *μ*Ci) of ^18^F-ML-8 in 0.2 mL sterile saline. Urine and blood samples were collected at 30 min separately. Blood was centrifuged (6000 rpm, 4 min) to separate plasma. Urine (10 *μ*L) and plasma (20 *μ*L) sample were injected into the analytic HPLC system.

### 2.6. Binding Assays with Apoptotic Jurkat Cells

To demonstrate the performance of ^18^F-ML-8 as a marker of apoptosis, it was used for detection of apoptosis induced by an anti-Fas antibody (CD-95) [20]. Jurkat cells (human adult leukemia T-cells, E6-1, Chinese Academy of Science, Shanghai, China) were cultured in Dulbecco's modified Eagle's medium with 10% fetal calf serum at 37°C in a humidified atmosphere containing 5% CO_2_. Cells at the logarithmic growth phase were harvested and adjusted to a concentration of 10^6^/mL in PBS, pH 7.5. Binding assays were performed as previously described [19, 21–23].

### 2.7. In Vivo Biodistribution

^18^F-ML-8 (0.74–1.48 MBq, 20–40 *μ*Ci) in 0.2 mL was administrated to adult female Kunming mice (5 per group). At 5 min, 30 min, 60 min, and 120 min after injection, mice were sacrificed by humane euthanasia, cervical dislocation. Blood was obtained through the eyeball, tissues and organs of interests were counted. All measurements were background-subtracted and decay-corrected to the time of killing and then averaged. Radioactivity values were calculated and presented as the percentage injected dose per gram (% ID/g) of tissue.

### 2.8. Small-Animal PET Imaging

In vivo PET imaging experiments were using the Inveon small-animal PET/computed tomography (CT) scanner (Siemens). Those allografted nude mice, treated and untreated, were imaged by PET/CT scanner after injection of 0.2 mL ^18^F-ML-8 solution (3.7–7.4 MBq, 100–200 *μ*Ci) via tail vein, after the mice were anesthetized with 5% chloral hydrate solution (6 mL/kg) and placed on a heating pad to warm the animal throughout the scanning. Imaging started with a low-dose CT scan, which was followed by a ten-minute PET scan. Ten-minute static PET images were acquired at four time points (30, 60, 90, and 120 min) after intravenous injection. The CT scan was used for attenuation correction and localization of the lesion site. Images were reconstructed by two-dimensional ordered-subsets expectation maximum (OSEM). For small-animal PET scan, regions of interest (ROIs) were drawn over the tumor and major organs on decay-corrected whole-body coronal images using Inevon Research Workplace 4.1 software. Radioactivity concentration (i.e., accumulation) of tumor or organs was obtained from the mean pixel values within the multiple ROI volume, which was converted into MBq/mL using a conversion factor. Assuming the density of tissue was 1 g/cm^3^, the ROIs were converted to MBq/g and then divided by the administered activity to obtain an imaging ROI-derived % ID/g.

### 2.9. Histologic Analysis

After the PET scans, SPCA-1 lung adenocarcinoma-bearing nude mice were euthanized and the tumor tissues (treated and untreated nude mice tumors) were collected for histologic analysis. Cell death was confirmed by the terminal deoxynucleotidyl transferase-mediated biotin-dUTP nick-end labeling (TUNEL) assay using a commercial kit (Promega, USA) according to the manufacture's protocol. After treatment, tumor sample slices were stained with fluorenscein-12-dUTP and 4′,6-diamidino-2-phenylindole (DAPI) for the observation of apoptotic and viable tumor cells, respectively. To ensure that our TUNEL assay correctly labeled apoptotic cells, we also collected positive and negative subsamples and monitored them for TUNEL signatures. Positive groups were treated by exposing fixed embryos to DNase (Promega, 1 U at 37°C) for 30 min. Negative controls were performed similar to the previous method without terminal deoxynucleotidyl transferase (TdT) treatment [24].

### 2.10. Statistical Analysis

Quantitative data were expressed as mean ± SD. Statistical analysis was performed using one-way ANOVA test version (SPSS 19.0). Statistical significance was defined as *P* < 0.05.

## 3. Results

### 3.1. Radiosynthesis

**Figure 2:**
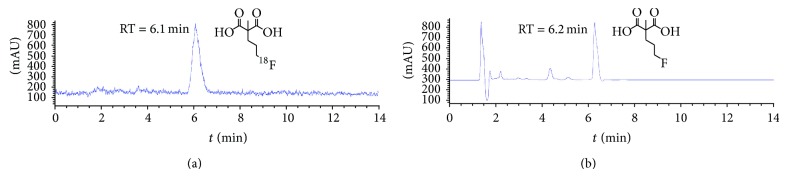
HPLC radioactive (a) and ultraviolet (b) chromatograms of the purified ^18^F-ML-8 solution coinjected with the standard (ML-8).

After detection, the radiolabeling and hydrolysis reaction was finished in 12 and 10 min, respectively. The overall uncorrected radiochemical yield of ^18^F-ML-8 was 21 ± 5% (*n* = 10) from ^18^F ion with a total synthesis time of 70 min. The specific activity was greater than 35.2 ± 19.5 GBq/*μ*mol (*n* = 10). The radiochemical purity was greater than 99% as analyzed by radio-HPLC (Figure 2). Coinjection of ^18^F-ML-8 and nonradioactive ML-8 assessed the identity of the radiopharmaceutical with a UV detection of 211 nm. Cold labeling of the precursor was performed in the TBAF-THF-CH_3_CN mixture (Figure 1(a)). Basic hydrolysis yielded the standard (ML-8) with no further purification.

### 3.2. In Vitro and In Vivo Stability

**Figure 3:**
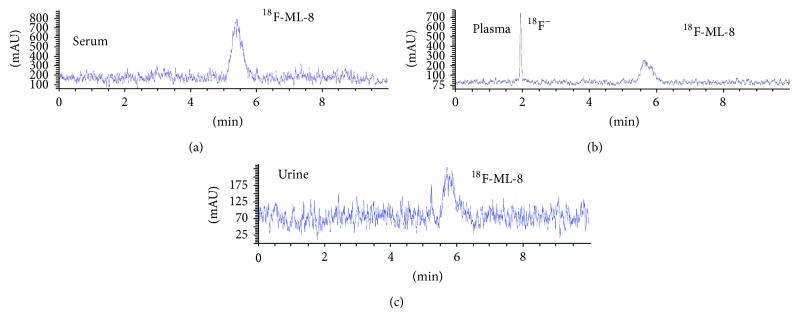
HPLC chromatograms of ^18^F-ML-8 in serum at 37°C 120 min after administration (a), in plasma (b) and urine (c) of normal mice 30 min after intravenous injection.

For the in vitro stability assay, ^18^F-ML-8 in serum was greater than 99% intact for 30 and 60 min and no less than 95% for 90 and 120 min (Figure 3(a)). For the in vivo stability study in plasma a little degradation of the product (around 35%) was noted for up to 30 min, which means only 65% of ^18^F-ML-8 kept intact (Figure 3(b)). In urine, the unchanged ratio was 30% up to 30 min (Figure 3(c)) and very low unchanged ratio for detection at 60 min.

### 3.3. In Vitro Cell Binding Assays of ^18^F-ML-8

The performance of ^18^F-ML-8 at targeting apoptotic cells was tested in vitro with Jurkat cells. The anti-Fas antibody treatment successfully induced Jurkat cells to undergo apoptosis. Compared to the control group (viable cells), there was a 4.2-fold increase in the uptake of ^18^F-ML-8 after the induction of apoptosis. In contrast, after inhibition of the apoptotic process by prior treatment with a caspase inhibitor (Z-VAD-FMK), increased uptake of ^18^F-ML-8 was not observed (Table 1). In addition, necrotic group cells did not manifest an obvious uptake of ^18^F-ML-8; the content of the radiolabeled probe shows a similar level to those in the control group (Table 1). These results demonstrate the selective uptake of ^18^F-ML-8 in apoptotic cells while not in the viable and necrotic cells and the effective inhibition to the apoptotic process by a caspase inhibitor.

### 3.4. Biodistribution Studies in Normal Mice

**Figure 4:**
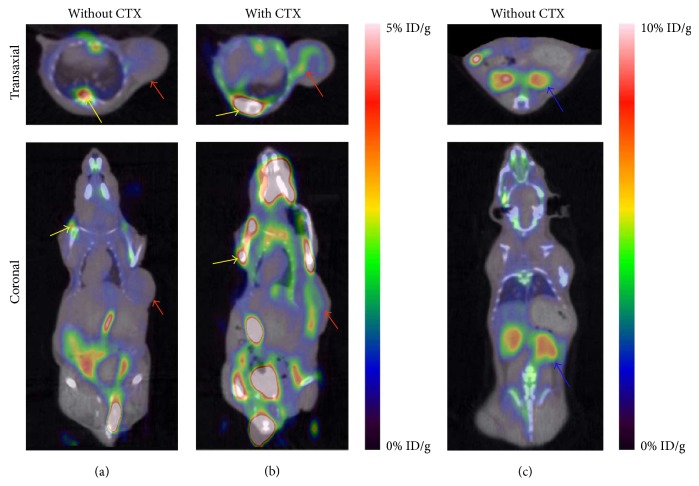
Representative images of the SPCA-1 tumor-bearing nude mice with CTX (intraperitoneal, 100 mg/kg). Small-animal PET images of the untreated (a) and treated (b) SPCA-1 xenograft obtained 1 h after ^18^F-ML-8 injection. Different slice of the PET images of untreated SPCA-1 tumor-bearing mice (c) (tumor is marked with red arrow; kidney is marked with blue arrow; fluorine signals accumulated in bone were indicated by yellow arrows).

^18^F-ML-8 distributed rapidly through the whole body after intravenous injection in healthy mice. And the probe rapidly accumulated and cleared from the blood (Table 2). The kidneys had the highest concentration at 30 min (24.5 ± 4.31% ID/g) after the injection and declined to a low level at 120 min (0.59 ± 0.25% ID/g). However, 30 min after injection, tissue with the highest level of radioactivity was the bone (7.57 ± 2.21% ID/g); the high level of radioactivity lasted to the end of experiment (120 min, 5.62 ± 0.77% ID/g). The rest tissues (such as brain, heart, lung, liver, and pancreas) experienced a relatively low uptake at 5 min and reduced gradually over the 120 min experimental assays. High radioactivity uptake level of the bone might indicate defluorination of the radiolabeled tracerin vivo. Imaging studies in normal and treated mice further confirmed the biodistribution results (Figure 4).

### 3.5. ^18^F-ML-8 PET Imaging

PET images and biodistribution data indicated that ^18^F-ML-8 was excreted primarily through urine-bladder system, reflected by remarkable accumulation in kidney and bladder (Figure 4(c)). Based on optimal uptake levels of radiopharmaecuticals in the major organs, we selected 1 h as typical PET images. Small-animal PET imaging was conducted on untreated (Figure 4(a)) and CTX-treated (Figure 4(b)) SPCA-1 human lung adenocarcinoma-bearing nude mice using ^18^F-ML-8. In addition, different slice of PET image was selected to prove the high accumulation of radioligand in kidneys (Figure 4(c)).

**Figure 5:**
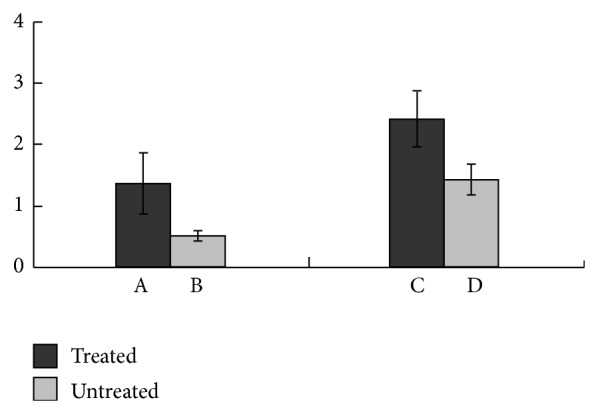
The uptake of ^18^F-ML-8 in treated (A) and untreated (B) SPCA-1 tumor xenografts at 1 h after injection. The tumor versus muscle (T/M) uptake ratio of ^18^F-ML-8 in treated (C) and untreated (D) tumor models 1 h after injection (*n* = 4; mean ± SD). The treated mice showed significantly higher tumor uptake of ^18^F-ML-8 and T/M ratio than the corresponding untreated mice. *P* < 0.05 compared with untreated group.

Apoptotic cell regions in tumors induced by CTX treatment were clearly visualized in the PET images performed with ^18^F-ML-8, compared to the images of other organs (muscle, brain, neck, and other organs, Figures 4(a) and 4(b)). In contrast, tumors that did not receive anticancer drug treatment had a low accumulation of radiolabeled probe. The tumor uptake of ^18^F-ML-8 in treated versus untreated SPCA-1 tumor models was 1.39 ± 0.51 versus 0.52 ± 0.09% ID/g; and the ratios of tumor versus muscle (T/M) in treated and untreated models were 2.46 ± 0.46 versus 1.45 ± 0.25, respectively (*n* = 4, Figure 5).

### 3.6. TUNEL Staining Assay

**Figure 6:**
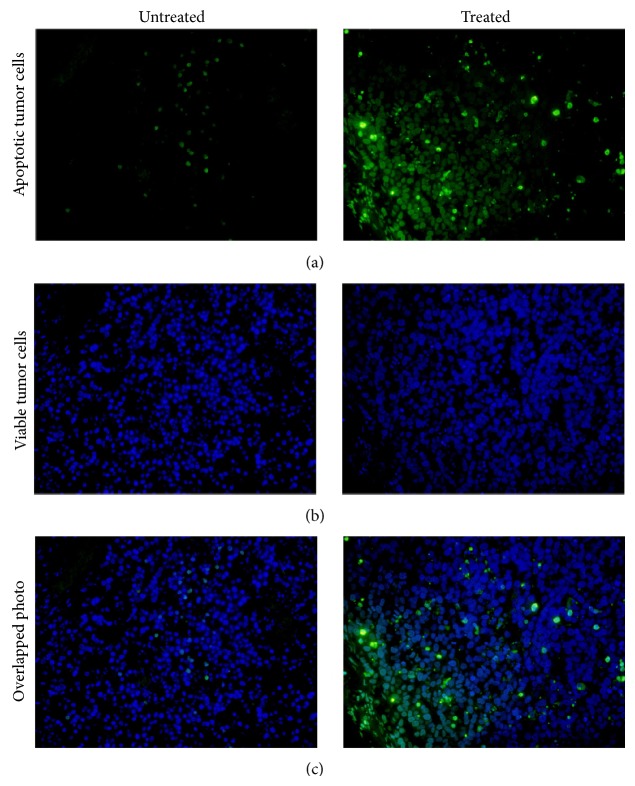
TUNEL staining analysis of untreated (left) and CTX treated (right) SPCA-1 tumor tissues. Seen under the fluorescence microscope at a certain wavelength, viable tumor cells were stained in blue (b), while the apoptotic tumor cells were stained in green (a). The overlapped photograph (c) indicated more apoptotic tumor cells (green color) in the treated tumor samples than the untreated tumor tissues.

For evaluation of the percentage of apoptotic cells in treated and untreated nude mice models, mice were sacrificed by cervical dislocation after scanning, and tumor issues were collected and performed with TUNEL assay to determine DNA damage. Sample slices were observed via the fluorescence microscope with specially appointed wavelength (*λ*) light according to different dyes. Apoptotic and live tumor cells were detected under the wavelength of 520 ± 20 nm and 460 nm, respectively. As shown in Figure 6, untreated samples with a relatively high proportion of viable tumor cells were stained with fluorescein-12-dUTP to give a blue color and barely green color. Obvious increased ratio of apoptotic cells was observed in the CTX-treated group samples, which were stained by DAPI to display a green color. The identity of the stained apoptotic cells was confirmed by positive and negative staining contrast (data not shown). Therefore, compared to the untreated group, CTX-treated tumor samples showed more apoptotic cells.

## 4. Discussion

Herein, we described the synthesis of ML-8 and its precursor, mesyloxy derivative, for radiolabeling with fluorine-18. The preparation of precursor was finished in five steps in the yield of 72%, much higher than that of ^18^F-ML-10's precursor (with the yield of 28%) [13]. ML-8 was obtained in two simple steps including fluorination and alkaline hydrolysis with the yield of 71%. The radiosynthesis procedure of ^18^F-ML-8 was performed using 18-Crown-6 and KHCO_3_ rather than Kryptofix 222 (K_222_) and potassium carbonate (K_2_CO_3_). The reason was that no radiolabeling product was detected by radio-HPLC analysis on the preliminary fluorination trials using K_222_-K_2_CO_3_ mixture. On the contrary, the mixture of 18-Crown-6 and KHCO_3_ gave stable and relatively good labeling yield (21 ± 5% non-decay-corrected). Considering factors that contribute to the stability of radiolabeling precursor itself, the weak alkali KHCO_3_ is more suitable than K_2_CO_3_ for the nucleophilic substitution. It means precursor was more stable and the yield was higher in KHCO_3_ than K_2_CO_3_ in CH_3_CN, which was in accordance with the experimental results.

^18^F-ML-8 was a useful and effective tool with extremely low molecular weight (MW 178) in detection of cell death. The structure was designed according to the reported PET tracer ^18^F-ML-10, a member of ApoSense family for apoptosis detection [14, 20, 25, 26]. The only difference between ^18^F-ML-8 and ^18^F-ML-10 was the carbon length of the side chains. ^18^F-ML-10 had a five-carbon side chain on the basis of malonic acid main body, while ^18^F-ML-8 had a three-carbon side chain. Apoptosis is a complicated process and is marked by its acidification of the outer leaflet and permanent externalization of the plasma membrane's inner leaflet. On the basis of this theory, ^18^F-ML analogues were designed and synthesized to selectively binding to and passing through the altered cell membrane and finally accumulating in the apoptotic cells.

Noninvasive detection of apoptosis could be useful tools in assessment of chemotherapy effect, as effective treatment usually results in apoptosis of cancer cells [27, 28]. We have previously reported the radiolabeling procedure and bioevaluation of the peptide PET tracer ^18^F-FPDuramycin [19]. Compared to the long synthetic procedure of ^18^F-FPDuramycin, it needs merely 70 min to get the desired product ^18^F-ML-8. The minimized molecular weight and structure enabled a high efficient radiolabeling process and fitted the large scale production in clinical trials (Figure 1(b)). Three simple synthetic steps including nucleophilic substitution of the precursor** 7**, hydrolysis in aqueous sodium hydroxide solution, and acidification yielded the desired product ^18^F-ML-8 with relatively high yield and purity.

Unlike the high stability of ^18^F-ML-10 in healthy rats reported in previous paper [20], radio-HPLC analysis showed that ^18^F-ML-8 in vitro (serum) had reasonable stability but a little defluorination in vivo of normal mice (Figure 3). The key factor that contributed to the distinct stability between ^18^F-ML-10 and ^18^F-ML-8 might be the length of side chain; a three-carbon side chain structure seems to be less stable than a five-carbon side chain compound. As a hexatomic ring is more stable and has lower energy level than an octatomic ring. Under alkaline condition, ^18^F-ML-8 presumably could be transferred to a more stable structure** 11** via the intramolecular cyclization reaction automatically (Figure S1 in Supplementary Materials), which might directly lead to the increased ^18^F ion density in alkalescent blood plasma and urine. In other words, the relatively high energy level of the proposed structure** 13** probably contributes to the superior stability of ^18^F-ML-10.

The in vitro cell binding assays showed visualized results of the selective accumulation of ^18^F-ML-8 in varied situation cells. In the anti-Fas antibody-dependent apoptosis tests, ^18^F-ML-8 was excluded from viable cells but revealed marked accumulation in cells undergoing apoptosis (the apoptotic status of anti-Fas treated Jurkat cells was confirmed by the treatment of Annexin V/PI Apoptosis Detection Kit and measured by the flow cytometry; see Figure S2 in Supplementary Materials). Unlike ^18^F-FPDuramycin binding to PE (phosphatidylethanolamine) on the extracellular plasma membrane of apoptotic cells, translocation of ^18^F-ML-8 seemed to have something to do with the intact, but depolarized, acidic and PS (phosphatidylserine) exposing plasma membrane. A possible transmembrane mechanism of ML-8 might be including three steps in the intact apoptotic cells: (1) acidification of the external membrane leaflet due to PS externalization facilitates proton capture by the malonate moiety, which resulted in increased hydrophobicity and facilitating penetration into the outer membrane layer; (2) transmembrane passage was facilitated by the depolarization, similar to other hydrophobic anions [29–31], and probably assisted by membrane scramblase activity; (3) sequestration in the cytoplasm is facilitated by the cytosolic acidification characteristic of apoptosis [19, 22]. However, this accumulation in apoptotic cells was blocked by caspase treatment, which resulted in uptake decreased to ground levels. In accordance with the proposed mechanism, background level uptake of ^18^F-ML-8 was detected in the necrotic cells group.

The biodistribution of ^18^F-ML-8 in normal mice showed a fast distribution into the whole body, which was corroborated by highest accumulation of radiotracer at 5 min after intravenous injection. Also it revealed a rapid elimination from blood and all the other organs, which was in accordance with previous results for ^18^F-ML-10 except for the bone [1]. ^18^F-ML-8 was excreted through the renal-urinary system, as kidney tissues accumulated ^18^F-ML-8 rapidly and excreted rapidly through the kidney and bladder, while the uptake of ^18^F-ML-8 in liver was quite low. The radiolabelled probe did not appear to cross the blood-brain barrier, as evidenced by low uptake in the brain throughout the studies. A little defluorination of ^18^F-ML-8 was observed in vivo, characterized by relatively high levels of radiotracers in bone until to the end.

In our PET imaging studies, selective higher uptake of ^18^F-ML-8 was observed in the CTX-treated SPCA-1 tumor compared with the untreated tumor models (Figure 4). High accumulation of ^18^F-ML-8 in kidneys further proved the radioligand was excreted via the renal-urinary system. The results demonstrated that ^18^F-ML-8 PET imaging enabled sensitive visualization of apoptosis induced by CTX treatment, but the clinical application might more or less be affected by the slightly instability in vivo. Therefore, ^18^F-ML-8 might not be a useful PET probe for the noninvasive monitoring of responses to anticancer chemotherapy. Whether tumor undergoing apoptosis or not was confirmed by TUNEL staining analysis (Figure 6), most cells in the untreated group were stained in blue (viable tumor cells), but green color cells (apoptotic tumor cells) took a greater percentage in the treated group.

## 5. Conclusion

In this study, we have successfully prepared the novel tracer ^18^F-ML-8 and examined its potential application in imaging of the tumor regions of CTX treated tumor-bearing mice. Furthermore, results of in vivo PET imaging, cell binding assays, and TUNEL assay suggest that ^18^F-ML-8 targets merely the apoptotic regions of tumors. The malonate motif structure of ML-8 is vital to the PET imaging of apoptosis in vivo; however, the length and structure of side chain still need to be optimized and discussed in the following study.

## Supplementary Material

Chemical synthetic procedures of the precursor, standard and some intermediates of ML-8 were shown in this supplementary materials, the flow cytometry result and a proposed mechanism of intramolecular cyclization were also included in this part.

## Figures and Tables

**Table 1:** Accumulation of ^18^F-ML-8 in Jurkat cells.

Experiment and cells	Mean ± SD % uptake/10^8^ cells	Treatment	Inhibition
Induction of apoptosis			
Control	0.126 ± 0.022		
Treated	0.529 ± 0.040^∗^	Anti-Fas antibody	
Inhibition of apoptosis			
Control	0.106 ± 0.007		Z-VAD-FMK
Treated	0.131 ± 0.038	Anti-Fas antibody	Z-VAD-FMK
Induction of necrosis			
Control	0.142 ± 0.080		
Treated	0.204 ± 0.004	Freeze-thaw	

Apoptosis was induced in Jurkat cells by treatment with anti-Fas, and necrotic cell death was induced by three thaw cycles. Each value is the mean ± SD % out of the three experiments, ^∗^ *P* < 0.01 compared with control group.

**Table 2:** Biodistribution of ^18^F-ML-8 in normal mice after intravenous injection.

Organ	5 min	30 min	60 min	120 min
Blood	7.41 ± 2.29	2.89 ± 0.61	1.04 ± 0.16	0.23 ± 0.10
Brain	0.34 ± 0.07	0.23 ± 0.14	0.10 ± 0.02	0.07 ± 0.04
Heart	4.27 ± 0.33	1.39 ± 0.35	0.64 ± 0.26	0.15 ± 0.07
Lung	5.61 ± 1.08	1.63 ± 0.41	0.77 ± 0.21	0.41 ± 0.17
Liver	3.80 ± 1.08	1.96 ± 0.37	1.21 ± 0.14	0.25 ± 0.07
Pancreas	2.98 ± 1.18	0.80 ± 0.25	0.47 ± 0.12	0.24 ± 0.10
Kidney	24.5 ± 4.31	7.10 ± 2.20	3.29 ± 1.12	0.59 ± 0.25
Spleen	3.22 ± 0.60	1.01 ± 0.25	0.38 ± 0.15	0.21 ± 0.12
Intestine	3.04 ± 0.27	1.06 ± 0.24	0.41 ± 0.07	0.21 ± 0.05
Muscle	2.68 ± 0.48	1.05 ± 0.11	0.39 ± 0.12	0.15 ± 0.06
Stomach	2.25 ± 0.26	0.81 ± 0.31	0.40 ± 0.19	0.19 ± 0.05
Bone	7.75 ± 1.99	7.57 ± 2.21	6.71 ± 1.50	5.62 ± 0.77

Note: data are mean % ID/g ± SD (*n* = 5).
