# Supplementary material for: Molecular PET Imaging of Cyclophosphamide Induced Apoptosis with 18F-ML-8
Source: Biomed Res Int. 2015 Apr 9;2015:317403. doi: 10.1155/2015/317403 (PMC4420799; doi:10.1155/2015/317403)
Supplement: Supplementary file 1 — Chemical synthetic procedures of the precursor, standard and some intermediates of ML-8 were shown in this supplementary materials, the flow cytometry result and a proposed mechanism of intramolecular cyclization were also included in this part. [file 317403.f1.pdf]

Supplementary Materials for

Molecular PET imaging of cyclophosphamide induced apoptosis with ^{18}F-ML-8

Shaobo Yao, Kongzhen Hu, Ganghua Tang*, Siyuan Gao, BaoguoYao, Caihua Tang, Dahong Nie, Ting Sun, Shende Jiang

* Correspondence author: E-mail: gtang0224@126.com

Chemical Synthesis

Preparation of 3-[(*tert*-butyldimethylsilyl)oxy]-1-bromopropane (3)

The synthetic procedure of precursor and cold labeling standard is shown in Fig. 1A. To a mixture of 3-bromo-propanol **2** (1 g, 7.2 mmol) and imidazole (1.18 g, 17.3 mmol) in anhydrous tetrahydrofuran (THF, 10 mL) was added a solution of TBDMSCl (2.39 g, 15.8 mmol) dissolved in anhydrous THF (10 mL) dropwise at -5 ºC over a period of 30 min. The reaction mixture was stirred at room temperature overnight under Ar (argon) protection. The reaction was monitored by TLC (ethyl acetate/light petroleum 1:4). The solvent was removed under reduced pressure and the residue was diluted with water and extracted three times with EtOAc. The organic layers were combined, washed with brine, dried (Na_2SO_4) and evaporated to obtain compound **3** as a colourless oil (1.77 g, 97 %). ^{1}H NMR ($CDCl_3$): δ 3.74 (t, 2H, *J*=6 Hz); 3.51 (t, 2H, *J*=6 Hz); 2.03 (p, 2H, *J*=6 Hz); 0.90 (s, 9H); 0.07 (s, 6H).

Preparation of Di-ethyl 2-[3-(*tert*-butyldimethylsilyloxy)-propyl]malonate (4)

Under Ar protection, sodium hydride (0.33 g, 8.3 mmol) was added slowly to a suspension of diethyl malonate (1.1 g, 6.9 mmol) in anhydrous THF (10 mL) at 0 ºC. The solution was stirred for 30 min at 0 ºC and 3-[(*tert*-butyldimethylsilyl)oxy]-1-bromopropane **3** (1.43 g, 5.6 mmol) dissolved in THF (10 mL) was added dropwise. The reaction mixture was stirred at room temperature for 1 h and further heated to 50 ºC for 4 h. The reaction was monitored by TLC (EtOAc/light petroleum 1:5). The solvent was removed under reduced pressure and the residue was diluted with water and extracted three times with EtOAc. The organic layers were combined, washed with Brine, dried (Na_2SO_4) and evaporated to yield product **4** as a colourless oil (1.65 g, 88 %). ^{1}H NMR ($CDCl_3$): δ

4.17-4,21 (m, 4H); 3.63 (t, 2H, *J*=6 Hz); 3.37 (t, 1H, *J*=7.2 Hz); 1.96 (m, 2H); 1.53-1.58 (m, 2H); 1.27 (t, 6H, *J*=7.2 Hz); 0.09 (s, 9H); 0.05 (s, 6H).

Preparation of Di-ethyl 2-[3-(*tert*-butyldimethylsilyl)-propyl]-2-methylmalonate (5)

Under Ar atmosphere, to a suspension of Di-ethyl 2-[5-(tert-butyldimethylsilyl)-propyl]malonate **4** (0.75 g, 2.3 mmol) in anhydrous THF (10 mL) sodium hydride (0.18 g, 4.6 mmol) was added slowly at 0 ºC. The mixture was stirred for 30 min at 0 ºC and a solution of CH_3Br (2.14 g, 23 mmol) dissolved in THF (10 mL) (cooled by ice water) was added slowly. The reaction mixture was stirred at room temperature for 1 h and further heated to 30 ºC for 2 h. The reaction was monitored by TLC (EtOAc/light petroleum 1:6). The solvent was removed under reduced pressure and the residue was diluted with water and extracted three times with EtOAc. The organic layers were combined, washed with Brine, dried (Na_2SO_4) and evaporated to give compound **5** as a colourless oil (0.78 g, 100 %). ^1H NMR ($CDCl_3$): δ 4.17 (q, 4H, *J*=7.1 Hz); 3.61 (t, 2H, *J*=6.4 Hz); 1.88-1.91 (m, 2H); 1.43-1.48 (m, 2H); 1.40 (s, 3H); 1.24 (t, 6H, *J*=7.1 Hz); 0.89 (s, 9H); 0.04 (s, 6H).

Preparation of Di-ethyl 2-(3-hydroxypropyl)-2-methylmalonate (6)

Di-ethyl 2-[5-(tert-butyldimethylsilyl)-propyl]-2-methylmalonate **5** (0.73 g, 2.1 mmol) was added to a mixture of TBAF (tetrabutylammonium fluoride, 1.1 g, 4.2 mmol) dissolved in THF (20 mL). The solution was stirred at room temperature overnight. The reaction was monitored by TLC (EtOAc/light petroleum 1:1). The solvent was removed under reduced pressure and the residue was diluted with water and extracted three times with EtOAc. The combined organic layers were washed with Brine, dried (Na_2SO_4) and evaporated to yield product **6** as a colourless oil (0.45 g, 92 %). ^1H NMR ($CDCl_3$): δ 4.18 (q, 4H, *J*=7.1 Hz); 3.64 (t, 2H, *J*=6.4 Hz); 1.92-1.94 (m, 2H); 1.51-1.56 (m, 2H); 1.42 (s, 3H); 1.25 (t, 6H, *J*=7.1 Hz).

Preparation of Di-ethyl 2-[(3-methylsulfonyloxy)propyl]-2-methylmalonate (7)

Under Ar protection, MsCl (methanesulfonyl chloride, 0.32 g, 2.8 mmol) diluted in anhydrous THF (10 mL) was added dropwise to a solution of Di-ethyl 2-(3-hydroxypropyl)-2-methylmalonate **6** (0.32 g, 1.4 mmol) and Et_3N (triethylamine, 0.42 g, 4.2 mmol) dissolved in anhydrous THF (10 mL) at 0 ºC. The solution was stirred at 0 ºC for 1 h and further at room temperature overnight. The reaction was monitored by TLC (EtOAc/light petroleum 1:1). The solvent was removed under reduced pressure and the residue was diluted with water and extracted three times with EtOAc. The

combined organic layers were washed with Brine, dried (Na_2SO_4) and evaporated to obtain product **7** as a colourless oil (0.42 g, 92 %). ^{1}H NMR ($CDCl_3$): δ 4.23 (t, 2H, J=6.3); 4.19 (q, 4H, J=7.1 Hz); 3.02 (s, 3H); 1.94-1.97 (m, 2H); 1.73-1.78 (m, 2H); 1.43 (s, 3H); 1.26 (t, 6H, J=7.1 Hz).

Preparation of Di-ethyl 2-(3-fluoropropyl)-2-methylmalonate (8)

Under Ar atmosphere, a TBAF solution in THF (1 M, 1.2 mL, 1.2 mmol) was added to a solution of mesylate **7** (0.22 g, 0.71 mmol) in anhydrous CH_3CN (acetonitrile, 15 mL) and the mixture was heated to reflux for 3 h. The reaction was monitored by TLC (EtOAc/light petroleum 1:4). The solvent was removed under reduced pressure and the residue was diluted with water and extracted three times with EtOAc. The combined organic layers were washed with Brine, dried (Na_2SO_4) and evaporated to yield product **8** as a colourless oil (0.14 g, 82 %). ^{1}H NMR ($CDCl_3$): δ 4.44 (dt, 2H, J=47.2, J=6.0); 4.19 (q, 4H, J=7.1 Hz); 1.96-2.01 (m, 2H); 1.64-1.78 (m, 2H); 1.42 (s, 3H); 1.25 (t, 6H, J=7.1 Hz).

Preparation of 2-(3-fluoropropyl)-2-methyl-malonic acid (9)

Di-ethyl 2-(3-fluoropropyl)-2-methylmalonate **8** (0.1 g, 0.43 mmol) was added to a mixture of KOH (1 M, 2.15 mL) and EtOH (ethanol, 2 mL), stirred at 50 ºC for 1 h. EtOH was evaporated under reduced pressure and HCl (hydrochloric acid, 1 M) was added to adjust the mixture to pH 5-6. After several times extraction with EtOAc, the organic solvent was collected and dried (Na_2SO_4) and evaporated to yield the standard ML-8 **9** as an offwhite powder (0.065 g, 86 %). ^{1}H NMR ($CDCl_3$): δ 4.43 (dt, 2H, J=47.3, J=6.0); 1.94-2.01 (m, 2H); 1.65-1.80 (m, 2H); 1.42 (s, 3H).

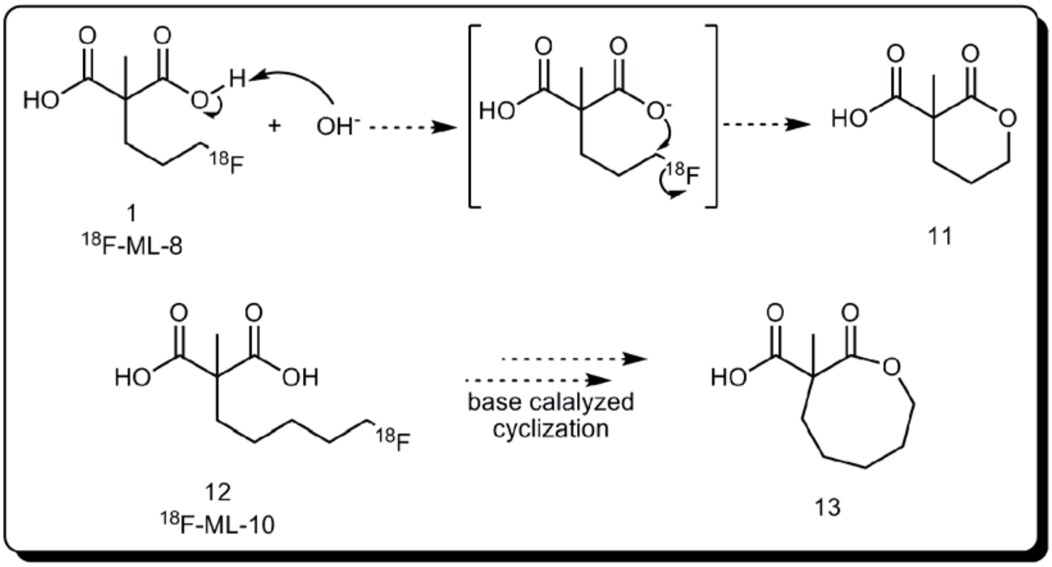

Fig. S1 A possible mechanism of intramolecularly dehydration of ^{18}F-ML-8 and ^{18}F-ML-10 to form a hexatomic and octatomic ring. The hexatomic ring structure **11** is more stable than octatomic ring structure **13**, meaning that octatomic ring in **13** is not easily formed

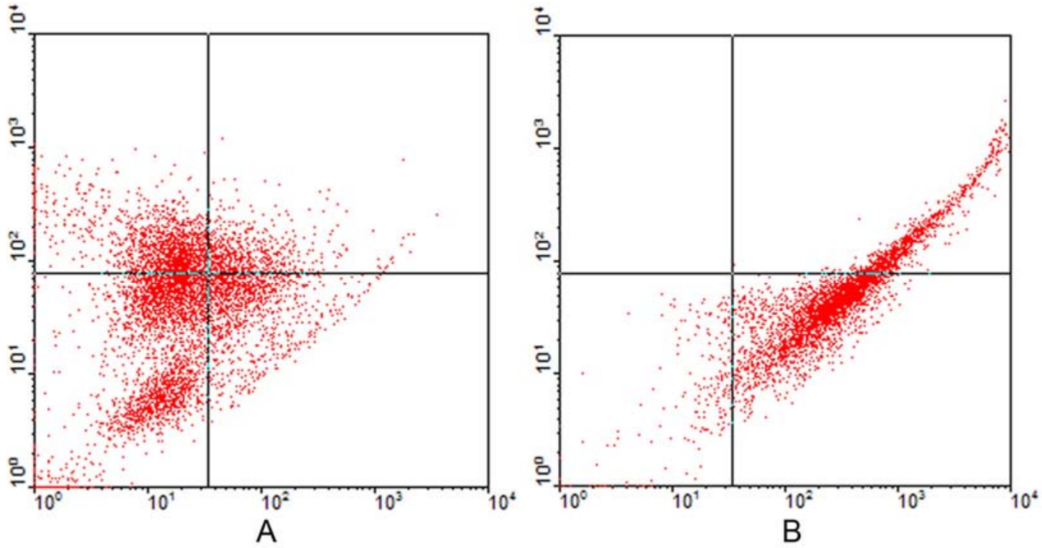

Fig. S2 Control and anti-Fas antibody treated Jurkat cells were performed according to the Annexin V/PI Kit protocol, and cells were measured by flow cytometry. Representative images of control (A) and apoptotic group (B) were presented. The percentage rate of normal (LL), apoptotic (LR) and necrotic (UR) cells in the control group were 48.8 %, 17.5 % and 9.85 % and in the apoptotic group the normal (LL), apoptotic (LR) and necrotic (UR) cells were 5.04 %, 71.0 % and 24.0 %, respectively. An obvious increase in the amount of apoptotic cells was observed in the apoptotic group, which means the anti-Fas antibody treated Jurkat cells did undergo apoptosis and ^{18}F-ML-8 was able to bind to apoptotic cells in vitro
